# Inertial sensor-based gait parameters reflect patient-reported fatigue in multiple sclerosis

**DOI:** 10.1186/s12984-020-00798-9

**Published:** 2020-12-18

**Authors:** Alzhraa A. Ibrahim, Arne Küderle, Heiko Gaßner, Jochen Klucken, Bjoern M. Eskofier, Felix Kluge

**Affiliations:** 1 Machine Learning and Data Analytics Lab, Friedrich-Alexander-University Erlangen-Nürnberg (FAU), Erlangen, Germany; 2 Computer Science Department, Faculty of Computers and Information, Assiut University, Asyut, Egypt; 3 Department of Molecular Neurology, University Hospital Erlangen, Erlangen, Bavaria Germany; 4 Fraunhofer Institut for Integrated Circuits, Erlangen, Bavaria Germany; 5 Medical Valley Digital Health Application Center, Bamberg, Bavaria Germany

**Keywords:** MS, Gait, Fatigue, Accelerometer, IMU, Machine learning, Digital biomarker

## Abstract

**Background:**

Multiple sclerosis (MS) is a disabling disease affecting the central nervous system and consequently the whole body’s functional systems resulting in different gait disorders. Fatigue is the most common symptom in MS with a prevalence of 80%. Previous research studied the relation between fatigue and gait impairment using stationary gait analysis systems and short gait tests (e.g. timed 25 ft walk). However, wearable inertial sensors providing gait data from longer and continuous gait bouts have not been used to assess the relation between fatigue and gait parameters in MS. Therefore, the aim of this study was to evaluate the association between fatigue and spatio-temporal gait parameters extracted from wearable foot-worn sensors and to predict the degree of fatigue.

**Methods:**

Forty-nine patients with MS (32 women; 17 men; aged 41.6 years, EDSS 1.0–6.5) were included where each participant was equipped with a small Inertial Measurement Unit (IMU) on each foot. Spatio-temporal gait parameters were obtained from the 6-min walking test, and the Borg scale of perceived exertion was used to represent fatigue. Gait parameters were normalized by taking the difference of averaged gait parameters between the beginning and end of the test to eliminate inter-individual differences. Afterwards, normalized parameters were transformed to principle components that were used as input to a Random Forest regression model to formulate the relationship between gait parameters and fatigue.

**Results:**

Six principal components were used as input to our model explaining more than 90% of variance within our dataset. Random Forest regression was used to predict fatigue. The model was validated using 10-fold cross validation and the mean absolute error was 1.38 points. Principal components consisting mainly of stride time, maximum toe clearance, heel strike angle, and stride length had large contributions (67%) to the predictions made by the Random Forest.

**Conclusions:**

The level of fatigue can be predicted based on spatio-temporal gait parameters obtained from an IMU based system. The results can help therapists to monitor fatigue before and after treatment and in rehabilitation programs to evaluate their efficacy. Furthermore, this can be used in home monitoring scenarios where therapists can monitor fatigue using IMUs reducing time and effort of patients and therapists.

## Background

MS is a disabling chronic disease affecting the central nervous system and leading to a variety of motor-symptoms and sensory impairments. It is caused by an autoimmune reaction against the myelin sheets of neurons resulting in relapsing and chronic disease progression [1]. MS symptoms can appear at any age but they were often initially observed in young adults. Most patients are diagnosed between the ages of 20 and 40 in the middle of their working lifespan and more than 2.3 million people all over the world have been diagnosed with MS [2]. Fatigue is considered one of the most common symptoms of MS, affecting about 80% of MS patients [1]. Additionally, MS patients reported fatigue to be the most irritating symptom [3] occurring at all stages of the disease [4]. It significantly affects functional capabilities of patients at both home and work, limiting daily activities and consequently reducing quality of life [5]. Previous studies showed that there is a strong association between symptomatic fatigue and muscle fatigue, impaired balance and motor function in MS patients [6, 7], in particular affecting the ability to walk which can be measured by gait analysis systems such as instrumented treadmills [8], camera-based systems [9, 10], or wearable sensors [11–13]. Predicting fatigue can help to evaluate treatment efficacy by monitoring and comparing fatigue before and after specific treatment programs. Gait and the ability to walk is a central part of everyday life activities. Thus, finding the relationship between fatigue and gait patterns can help the therapist to develop suitable rehabilitation strategies for reducing the impact of fatigue on MS patients [14]. Furthermore, fatigue was found to strongly affect fall risk, balance performance, and fear of falling [15]. Hence, interventions to reduce fatigue can contribute to decreasing fall risk and fall-related injuries and improving overall quality of life.

### Fatigue assessment in MS

Only few studies focused on the assessment of fatigue from gait patterns in MS patients. Kalron [8] studied the association between fatigue in MS patients as reported by the Modified Fatigue Impact Scale (MFIS) and gait parameters based on an instrumented treadmill. The author observed that perceived fatigue is related to specific gait parameters. However, as some patients cannot walk on a treadmill without assistance, not the whole spectrum of patients could be assessed. Furthermore, treadmill walking differs from overground walking and therefore leads to additional variability in gait patterns [16].

Moreover, McLoughlin et al. [9] performed gait analysis in MS patients with moderate disease severity (3–6) as measured by Expanded Disability Status Scale (EDSS) using a marker-based system to find the changes in gait following the Six-Minute Walking Test (6MWT). They observed a significant increase in fatigue after the 6MWT. They reported no effect on spatio-temporal gait parameters but found significant effects on some kinetic and kinematic parameters, such as a decrease in ankle dorsiflexion and increase in knee and hip flexor moments and hip power absorption. Crenshaw et al. [10] also used a camera-based system for gait analysis in patients with an average (± SD) EDSS of 3 (± 1) points. The patients performed 10-m walking without assistance and repeated the test three times (morning, afternoon, and 1 week later in the morning). In contrast to the previous study, no changes in kinetic or kinematic parameters between fatigued and non-fatigued MS patients were observed.

Sacco et al. [17] used a gait-mat (GAITRite) to compare gait parameters and changes in fatigue (as measured by Wuerzburg Fatigue Inventory for Multiple Sclerosis) between MS patients and healthy controls during inpatient rehabilitation. They found a correlation of physical fatigue with gait velocity, cadence, and stride length.

The limitations of the previously described studies are related to the obtrusive and stationary systems that require high efforts in data acquisition and analysis. Gait patterns obtained might also not be representative for real world walking due to the constraints applied on patients.

### Inertial wearable sensors for gait analysis

For studying the effects of neurological diseases on gait, inertial sensors are reliable, portable and low-cost tools that can be used for analyzing gait both in clinical or home settings [18]. Wearable sensors have already been used for gait analysis in different clinical applications. Previous studies reported the clinical validity of IMU based gait analysis and its feasibility to measure spatio-temporal gait parameters in Parkinson disease [19, 20] and atypical parkinsonian disorders [21, 22]. Also sensor-based gait analysis was used to measure impairment and disease severity in Huntington’s disease [23, 24]. Moreover, Moufawad El Achkar et al. [25] analyzed gait in elderly people using shoes instrumented with IMU and pressure sensors. They reported the validity of foot worn sensors in monitoring and classifying daily activities of old people. Flachenecker et al. [26] validated the use of wearable sensor-based gait analysis in MS. They detected correlations between IMU based gait parameters and disease severity as measured by EDSS and proved the ability of IMUs to detect differences in gait parameters between healthy controls and MS patients even in earlier stages of the disease. Additionally, Angelini et al. [27] analyzed gait in MS patients using wearable sensors and compared gait parameters obtained in two different clinical settings. They reported the consistency of gait parameters in different settings and herewith confirmed the reliability of wearable sensors in gait analysis.

### Inertial wearable sensors for fatigue assessment in MS patients

Motta et al. [11] investigated the feasibility of wearable sensors for gait analysis of MS patients. They used seven IMUs placed on patient’s pelvis, thigh, shank and foot. They presented a new index that measured range of motion (ROM) of leg joints and classified patients in fatigued and non-fatigued states based on this index with an accuracy of 80.7%. A further study by Taborri et al. [12] aimed at evaluating changes in walking parameters of MS patients due to fatigue by comparing them with a healthy control group during the 6MWT. Using seven sensors, they found that at the last minute of the test when patients were fatigued most, the ROM decreased in all lower limb joints of patients compared with healthy controls. Also, Morris et al. [13] studied the relation between gait and self-reported fatigue in MS over the day using foot pressure sensors. Patients performed four trials of 10-m walking at the morning and afternoon and average gait parameters were calculated. They observed that patients reported higher fatigue in the afternoon than in the morning but gait patterns remained consistent over the day indicating that there is no relation between self-reported perceived fatigue and gait.

To the best of our knowledge, no studies have been conducted to estimate the degree of fatigue in MS patients using only foot-worn IMUs obtained from longer gait bouts during overground walking which is an unobtrusive way of measuring gait. Studying the relationship between fatigue and IMU-based gait parameters can give an insight to clinicians about gait patterns of patients with different fatigue levels and which gait parameters can be considered as digital biomarkers for fatigue. Moreover, this can be used in potential home-monitoring scenarios where a patient stays at home wearing foot sensors and relevant measures that can be used in management or evaluation of specific therapies or treatment programs are sent to clinicians, thereby reducing time and effort of both patients and clinicians.

Thus, the aim of this study is to investigate the relationship between patient-reported fatigue and spatio-temporal gait parameters as measured by foot-worn IMUs, analyze the change of gait parameters over the course of a 6MWT, and to predict the degree of fatigue in MS patients using IMU sensors by perceived exertion after the 6MWT. The result of this study is a step towards the implementation of unobtrusive, portable, and low-cost systems for MS fatigue assessment in real-world walking environments.

## Methods

### Participants

Data was collected in the MS center of the University Hospital Erlangen (Germany) and the Neurological Rehabilitation Center Quellenhof (Bad Wildbad, Germany). The study included forty-nine patients diagnosed with MS, 32 women and 17 men aged 41.6 (SD ± 10.4) (Table 1). Patients were included if they were older than 18 years and their EDSS was below 7 (e.g. they were able to walk for at least 10 m) [26]. All subjects gave their written informed consent for inclusion before they participated in the study. The study was conducted in accordance with the Declaration of Helsinki, and the protocol was approved by Medical Faculty, Friedrich-Alexander University Erlangen- Nürnberg (Re.-No. 4208).

**Table 1:** Patients characteristics

N	49
Sex (m:f)	17:32
Age (years)	41.6 ± 10.4
EDSS (median/range)	3.7 (1–6.5)
No. of patients with specific disability level	
Moderate disability (EDSS < 4)	28 (57%)
Significant disability (EDSS ≥ 4)	21 (43%)

### Perceived fatigue

Borg scale of self-perceived exertion was used as a measure of fatigue [28]. It is a categorical scale measuring self-reported exhaustion of patients during exercise (Table 2). It ranges from 6 (no exertion) to 20 (maximum exertion). The notion of fatigue was added to the definition of Borg by Noble et al. [29] and emphasized by the American College of Sports and Medicine [30]. The Borg scale has been reported to be a good estimator of muscle fatigue after exhaustive tasks [31, 32]. Moreover, it was validated to be a reliable outcome in the fatigue assessment of MS patients [33]. During the performance of the 6MWT, the patient was asked every minute to rate exertion on the Borg scale. The maximum measured value was used as representative fatigue level for the 6MWT.

**Table 2:** Borg scale of perceived exertion [28]

Description of exertion	Borg rating
None	6
Extremely light	7–8
Very light	9–10
Fairly light	11–12
Somewhat hard	13–14
Hard	15–16
Very hard	17–18
Extremely hard	19–20

### Gait test and spatio-temporal gait parameters

One IMU (tri-axial accelerometer and gyroscope, SHIMMER 3 sensor, Shimmer Research Ltd., Dublin, Ireland) was attached to the lateral side of the ankle of each shoe [34]. Data was recorded at a sampling rate of 102.4 Hz, stored on a tablet and then transferred and analyzed on a computer. Patients performed a 6MWT in which they walked back and forth for 6 min on a straight 20 m long path at their own speed. This test is reliable, feasible, and highly recommended for use in MS patients [35–37]. Stride segmentation was performed using multi-dimensional subsequence dynamic time warping proposed by Barth et al. [34]. Stride-wise parameters (stride time, stride length, swing time, stance time, gait velocity, toe-off angle, heel-strike angle, maximum toe clearance, and maximum lateral excursion) were calculated using the parameter detection method proposed by Rampp et al. [38].

**Fig. 1:**
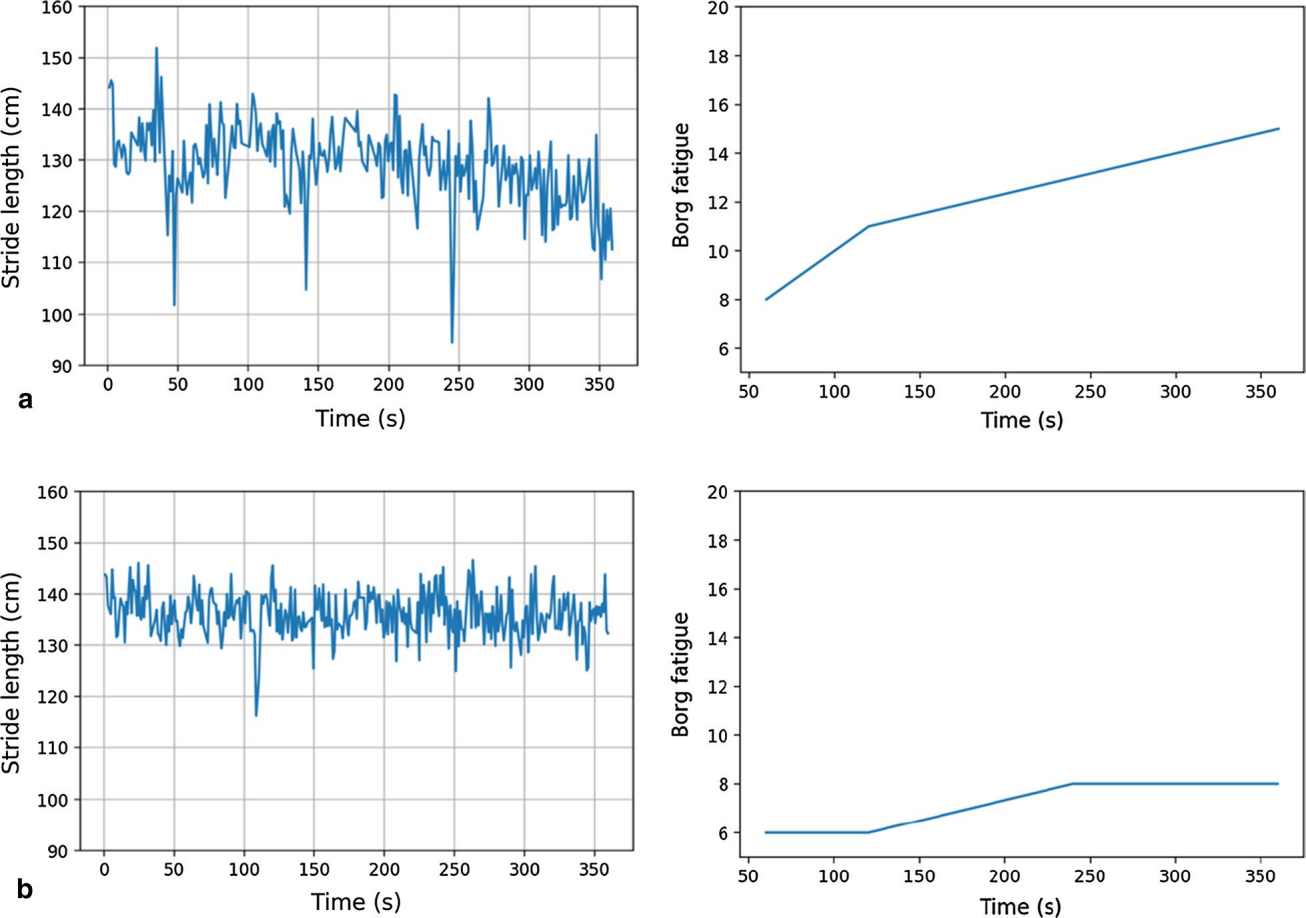
Change of stride length and fatigue level over 6-min-Walk-Test (6MWT) for two patients: **a** patient with fatigue rate 15 on Borg at the end of 6MWT; **b** patient with fatigue rate 8 at the end of 6MWT

To minimize the effect of inter-individual differences such as body weight, length, and general ability to walk, relative gait parameter changes were assessed by applying a normalization. This was performed by taking the difference in mean gait parameters between the empirically chosen first and last seventy strides of the test. Different numbers of strides were tested for performing normalization and the number of strides that gave the higher prediction accuracy was chosen. As an example, the baseline stride length from two patients with different fatigue levels is already different (Fig. 1). Additionally, the change of stride length over the test time is varying between the two patients which means that it might be used as a biomarker for fatigue assessment and can have more discriminative power than using the mean value of the absolute gait parameter.

### Regression analysis

For predicting fatigue, a regression analysis was performed using fatigue based on the Borg scale as dependent variable and combinations of normalized gait parameters as independent variables. Principal component analysis (PCA) was performed on the whole dataset to transform the normalized parameters to components with significant amount of variation contained within the dataset which help to reduce type I error. Principal components that explained the largest amount of variance (more than 90%) within the whole data were used for training our model. Afterwards, based on those principal components as input, a regression analysis using a Random Forest regressor was performed to estimate the Borg value. The model was trained and validated using a nested 10-fold cross validation. For hyper-parameter tuning, a randomized grid search was performed with hyper-parameters ranges as following: number of estimators from 200 to 22,000 with steps of 10; maximum depth from 10 to 100 with steps of 11; minimum number of samples to split were (2, 5, 10 to 20); and minimum number of samples at a leaf node were (1, 2 and 4). Furthermore, the correlation between fatigue and EDSS score was calculated using Spearman correlation.

## Results

**Fig. 2:**
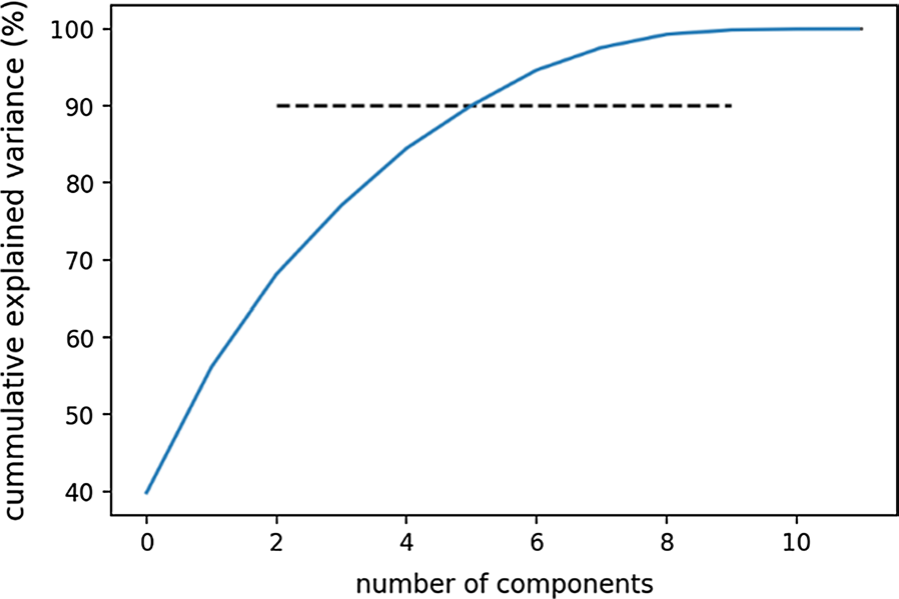
Variance explained by PCA (the horizontal line represents the threshold for selecting the number of components)

Six principal components were used that explained more than 90% of the variance within the dataset (Table 3, Fig. 2). These components were used as independent variables in a Random Forest regression model. The fatigue measured on the Borg scale was used as dependent variable. The mean absolute error was 1.38 ± 1.07 on the Borg scale. To see the effect of normalization, the same analysis was performed using non-normalized gait parameters by calculating the mean parameters and using them as input to PCA and regression analysis. The mean absolute error was 1.69 ± 1.03. Additionally, regression analysis was performed using normalized parameters directly as input without using PCA and the mean absolute error was 1.62 ± 1.04.

**Table 3:** Contributions of normalized gait parameters to principle components

Component	Highly contributed gait parameters
1	Stride length, gait velocity, stride time
2	Maximum toe clearance, stride time
3	Heel strike angle, stride length
4	Turning angle
5	Heel strike angle, toe off angle, maximum lateral excursion
6	Max toe clearance, turning angle

**Fig. 3:**
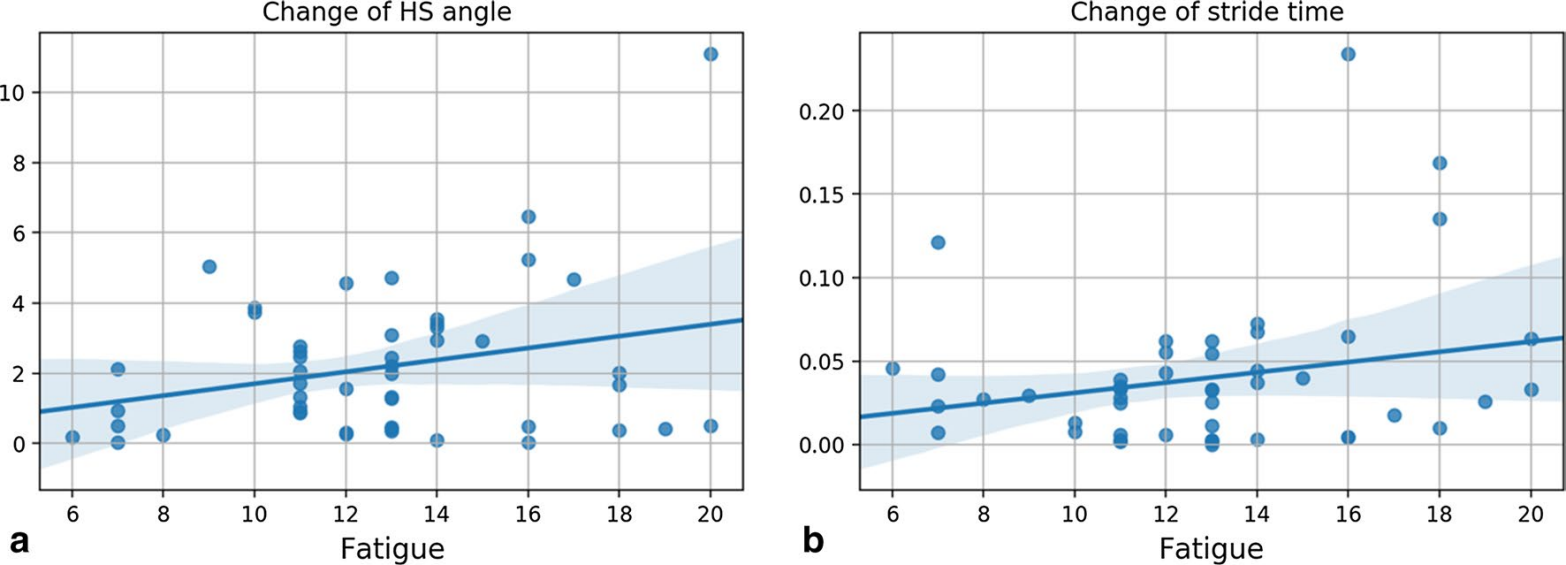
Change of **a** HS angle and **b** stride time over different levels of fatigue

From our model, the most important components that had high contributions to the decisions made by Random Forest regressor were the 2nd and 3rd components that consisted mainly of normalized stride time, maximum toe clearance, heel strike angle, and stride length. This indicates that there is a relation between fatigue and these gait parameters. For example, by plotting the change of HS angle and stride time from different patients over fatigue levels (Fig. 3), we can notice significant correlation between fatigue and these parameters.

**Fig. 4:**
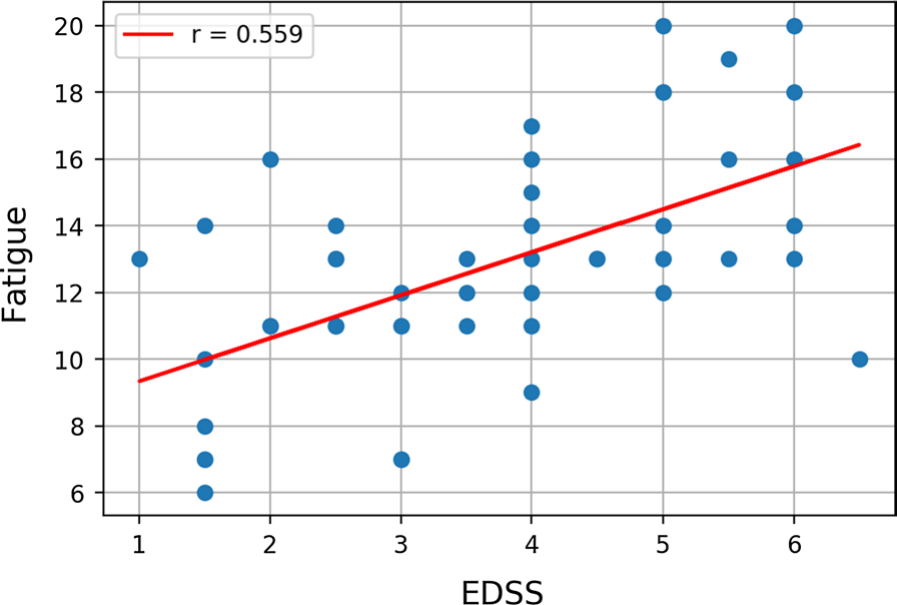
Correlation between fatigue and EDSS

Additionally, the correlation between fatigue and EDSS was moderate (Fig. 4) implying that EDSS may not predict fatigue levels adequately and that higher EDSS may not necessarily cause fatigue.

## Discussion

The aim of this study was to explore the relationship between gait parameters as measured by wearable sensors and fatigue assessed on the Borg scale in MS patients. Only foot worn sensors were used that are easy to adopt and suitable for unobtrusive use in real-world environments in addition to their economic benefits. All gait parameters were normalized to remove the effect of individual patient characteristics such as body mass and length. Gait parameters changed by the end of the test which could be a result of muscle fatigue that appeared after an exhaustive test. This change is related to the highest fatigue level reached by the end of the test. This was also reported by Sehle et al. [39] who found changes in gait patterns following an exhaustive walking due to muscle fatigue.

Regression analysis was performed to predict the level of fatigue. The mean absolute error of 1.38 represents one level on Borg scale. As the exhaustion categories on the Borg scale span two points (Table 2), our method allows an accurate prediction of the correct maximum exhaustion category. The resulting error might be caused by inter-individual differences in effort estimation as some people may overestimate or underestimate their actual fatigue level [40]. Using non-normalized gait parameters, the mean error was high resulting in lower overall accuracy which means that the model had difficulty in learning and extracting patterns from non-normalized data. This could be due to variability between participants in anthropometric characteristics. Thus, it was necessary to normalize gait parameters. This was also supported by previous studies that used normalization for gait parameters either by using body height or mass or a combination of them [41–43] to overcome this problem. They reported that the variance in gait parameters was reduced significantly after normalization. However, we used normalization based on calculating the difference in gait parameters between the first and last strides because weight and height were not collected in our dataset. This type of normalization represents dynamic changes over time and using those changes as input for the prediction model. Additionally, this eliminated individual differences by representing change of gait parameters instead of using absolute values. One challenge with normalized gait parameters was the need for selecting a specific number of strides at the beginning and end of the test and testing the accuracy of the model using different number of strides. A heuristic search was performed to select the number of strides that gives the best prediction accuracy.

Our model revealed that there is a strong relation between fatigue and some gait parameters (normalized stride time, maximum toe clearance, heel strike angle, and stride length). Similarly, Sacco et al. [17] found significant correlations between physical fatigue and stride length (− 0.50) and velocity (− 0.54). Furthermore, this was also reported by Karlon [8] who reported a significant correlation between fatigue and stride length (− 0.32) in moderately severe patients (EDSS between 3.5 and 6.5). However, instrumented treadmills as used by Sacco et al. do not represent real-world walking [44] and affect gait variance as compared with overground walking [45]. In contrast, results provided from camera-based systems [9] contradict our results as McLoughlin et al. [9] observed no significant correlation between spatio-temporal gait parameters and fatigue whereas some kinetic and kinematic changes were found after the 6MWT. However camera-based gait analysis systems have restrictions related to walking space and inability to monitor real-world walking [46]. Furthermore, systems depending on cameras and marker tracking exhibit high operational costs which consequently restrict their use in clinical applications [47]. Regarding pressure sensors, no correlations were found between fatigue and gait parameters [13]. This could be due to performance limitations of pressure sensors which may result in false predictions of peak pressure [44].

Additionally, the relation between EDSS and fatigue was moderate which means that fatigue may occur at any stage of the disease and may not be a result of severity of the disease. Also, previous studies reported weak correlation between fatigue and EDSS recommending that fatigue should be investigated regardless of severity status of MS patients [48, 49].

Limitations of our study include the use of only a small dataset. More data set is needed to ensure generalizability of our model to the whole disease population. Another limitation is that other factors affecting fatigue are not taken in consideration with the Borg scale such as sleeping disorders, depression and other cognitive impairments. Also, patients need to be instructed first about how to estimate their perceived effort on the Borg scale accurately. Furthermore, other fatigue measurement scales and their correlation with Borg need to be investigated. However, the relation between overall fatigue and fatigue related to specific exhausting tasks was investigated in a previous study that reported a significant association between overall fatigue and some muscle fatigability measures induced from exhaustive tasks [50, 51]. Additionally, our study is a cross-sectional study which did not measure fatigue longitudinally, which could yield valuable information about disease progression.

For future studies, prediction accuracy can be improved by using larger datasets and applying deep learning approaches because of its promising results in many healthcare applications and their reliability for using in gait analysis [52]. Larger datasets can be collected by incorporating data from home monitoring or combining data obtained from multiple hospitals with the evidence that there are no significant differences between IMU data collected in completely different settings [27]. Furthermore, measuring gait parameters in real-world environments may give different and more meaningful measures such as walking bout length that could give reliable clinical information as found by Storm et al. [53]. Also, a longitudinal study can be adopted in future work to predict progression of fatigue over a long period using home monitoring tools so that suitable fatigue treatment programs can be evaluated. Additionally, monitoring fatigue can also be used for assessing and monitoring effectiveness of treatments and rehabilitation programs due to the feasibility of wearable devices as monitoring tools [54]. Furthermore, investigating other fatigue objective measures can help to remove dependency on patients for assessing their fatigue level and can give more accurate prediction.

## Conclusions

The use of wearable sensors in gait analysis with MS patients is still an open research field. Previous studies using different gait analysis systems showed different and sometimes contradicting relationships between fatigue in MS and gait parameters. Thus, there was the need of studying the relationship of fatigue and gait parameters in more realistic walking scenarios. Wearable sensors allow gait analysis during unconstrained, continuous and longer overground walking bouts. We observed an association between self-perceived fatigue and spatio-temporal gait parameters of MS patients by the end of an exhaustive task such as the 6MWT. The system may be used by clinicians as a monitoring tool in their treatment and intervention programs to reduce the degree of fatigue in MS patients. Potentially, this can be used for assessing the feasibility of different rehabilitation and treatment programs in both clinical or home environments. Furthermore, the unobtrusive system may be used to monitor fatigue longitudinally in large cohort studies and additionally taking other objective fatigue measures into consideration.

## Data Availability

The datasets used and/or analyzed during the current study are available from the corresponding author on request.

## Abbreviations

MS: Multiple sclerosis
IMU: Inertial Measurement Unit
MFIS: Modified Fatigue Impact Scale
6MWT: Six-Minute Walking Test
ROM: Range of motion
EDSS: Expanded Disability Status Scale
PCA: Principal component analysis
